# Phylogeny and Specific Determination of *Gloydius halys-intermedius* Complex Based on Complete Mitochondrial Genes

**DOI:** 10.3390/genes16030276

**Published:** 2025-02-25

**Authors:** Lijie Jin, Zuyao Xia, Ning Liu, Shengyue Hou, Chuandong Lv, Lianyou Tang, Shuguang Feng, Jingsong Shi, Ming Bai

**Affiliations:** 1 Key Laboratory of Animal Biodiversity Conservation and Integrated Pest Management (Chinese Academy of Sciences), Institute of Zoology, Chinese Academy of Sciences, Beijing 100101, China; jinlijie@ioz.ac.cn (L.J.); liun@ioz.ac.cn (N.L.); 2 University of Chinese Academy of Sciences, Beijing 100049, China; 3 Department of Evolution, Ecology & Biodiversity, University of California, Davis, CA 95616, USA; bxia@ucdavis.edu; 4 Department of Wildlife, Fish and Conservation Biology, University of California, Davis, CA 95618, USA; hmdhou@ucdavis.edu; 5 National Nature Conservation of Snake Island and Laotieshan Mountain, Dalian 116041, China; lvchuandonglv@163.com (C.L.); 15140571392@163.com (L.T.); yanglao-fengshuguang@foxmail.com (S.F.); 6 Institute of Vertebrate Paleontology and Paleoanthropology, Chinese Academy of Sciences, Beijing 100044, China; 7 Northeast Asia Biodiversity Research Center, Northeast Forestry University, Harbin 150040, China

**Keywords:** mitochondrial genomes, Asian pit viper, infraspecific taxonomy

## Abstract

**Background**: The phylogenetic resolution within the *Gloydius halys-intermedius* Complex remains debatable due to the following reasons: loci selection in previous studies varied between authors; limited dataset (1−5 mitochondrial or nuclear gene fragments); lack of sampling density; and nodal supports at specific nodes remain weak, specifically within *Gloydius cognatus*, *G. halys*, and *G. stejnegeri*. **Objectives**: To revise the taxonomic and phylogenetic relationships within the *G. halys-intermedius* Complex, we reconstructed the molecular phylogeny and performed species delimitation based on the complete mitochondrial genomes. **Methods**: In this study, twelve nomenclatural groups of *Gloydius* species were involved in the computation of Bayesian phylogenomic inference, five of the twelve nomenclature groups were newly sequenced, while the rest were acquired from the National Center for Biotechnology Information (NCBI). The Bayesian phylogenomic inference was constructed based on 13 mitochondrial protein-coding genes. Species delimitation was performed by two distance-based methods (ABGD and ASAP) and two tree-based methods (GMYC and bPTP). **Results**: This research resolved the systematic relationship within the *G. intermedius* Complex with the support of mitogenome-based phylogenomics, while indicating cryptic diversity within the *Gloydius halys-intermedius* Complex: *G. intermedius* samples from South Korea show as paraphyletic to the cluster of the samples from northeastern China. Species delimitation results based on four models resemble each other, supporting *Gloydius caucasicus*, *G. cognatus*, *G. halys*, and *G. stejnegeri*, each representing full species. The species delimitation results of this research also resemble the nomenclatural species based on previous morphometrical results. This research indicates that species delimitation efforts based on the phylogenomic approach would likely resolve complex evolutionary relationships.

## 1. Introduction

The Asian pit vipers, genus *Gloydius* Hoge & Ramano-Hoge, 1978, a group of small-bodied anterior-fanged snakes, widely distributed from northeastern to central Asia. This genus contains more than 23 specific-leveled clades spread throughout diverse habitats from temperate forests at lower elevations to alpine meadows at higher elevations, such as the Qinghai–Tibetan Plateau [1,2,3,4]. Morphometrics and previous molecular phylogenetics conducted in this genus indicate three intragenic lineages: the *G. blomhoffii* Complex, *G. intermedius-halys* Complex, and *G. strauchi* Complex [5,6].

The current molecular phylogenetics inference has resolved the systematic relationship at specific levels under both the *G. blomhoffii* Complex and *G. strauchi* Complex with strong nodal support [2,3,4,7,8,9,10]. However, due to complex overlapping distributions, possible interspecific gene flows, and less genomic data involvement within the *G. intermedius-halys* Complex, the evolutionary relationships and the species delimitation under this species complex remain debatable [5,11,12,13]. In the molecular phylogeny of Sino-distributed *Gloydius* species constructed by Xu et al. [14], the two taxa labeled as “*G. intermedius*” did not display monophyletic topology [14], and were subsequently clarified as misidentifications of specimens of the two different subspecies under *G. halys*. Shi et al. [13] initially reconstructed the molecular phylogenetics of the *G. intermedius-halys* Complex with combined mtDNA ND4 and Cyt *b* [13], indicating *G. halys* and *G. stejnegeri* each represents a valid taxon at specific level. This taxonomic conception has been accepted by most studies [1,2,3,11,15,16]. However, in several recent publications, the diversity of the *G. intermedius-halys* Complex is still underestimated, these species are still conflated as *G. halys* without illustrations or reliable data support [12,17,18].

In previous studies, little genomic data were used to construct molecular phylogeny compared to the accumulating sampling and sequencing strength in recent years. Previous data usually contains 2–5 mtDNA gene sequences or a combination of both mtDNA and nucDNA [1,2,3,4,13,14,16,19]. As a result, some nodal supports remain at questionable levels.

Recent studies on molecular species delimitation in reptiles and insects [20,21,22] have provided new approaches for resolving taxonomic problems at specific level. However, these efforts have not been applicated to Asian pit vipers yet. To provide higher-resolution molecular phylogenomic inference within the *G. intermedius-halys* Complex, with the support of accumulated genomic data and strong sequencing strength, multiple mtDNA genomes of the Sino-distributed *Gloydius* species were acquired. Hence, to further investigate the inter-specific evolutionary relationship within the *G. intermedius-halys* Complex, an initial phylogenomic inference and molecular species delimitation of *Gloydius* species were performed in this study.

## 2. Materials and Methods

### 2.1. Samples and DNA Extraction

**Figure 1:**
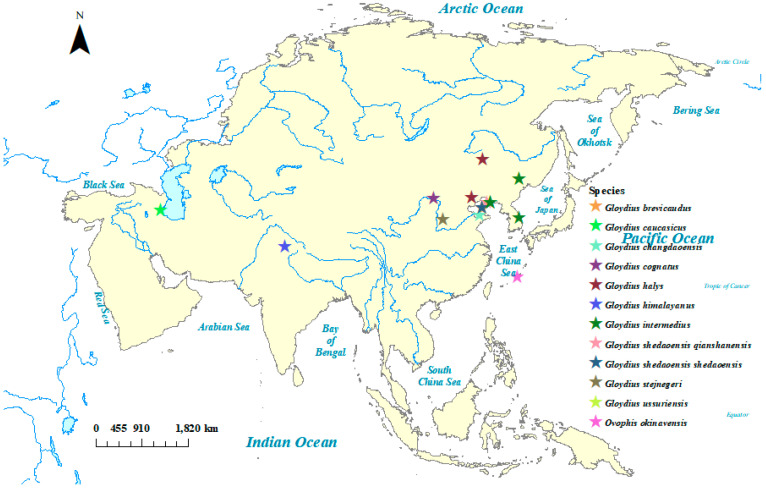
Distribution of molecular samples in this study. Each star represents a species of *Gloydius*.

In this study, five taxa of *Gloydius* were sampled. The detailed specimen information is listed in Table 1. In addition, the mitochondrial genome of seven *Gloydius* species were downloaded from GenBank for phylogenomic analysis. The distribution map of *Gloydius* in this study was drawn with ArcGis (Figure 1). *Ophis okinavensis*, a close relative of *Gloydius*, was selected to be the outgroup. Liver tissues were dissected to extract the whole genome using a TGuide Smart Universal DNA Kit (TIANGEN, Beijing, China) with the TGuide S16 Nucleic Acid Extractor. And the whole genome sample was deposited in a refrigerator at −20 °C at the Institute of Zoology, Chinese Academy of Sciences (IOZ, CAS).

### 2.2. Genome Sequencing, Assembly, and Annotation

The genomes were sequenced by the Illumina HiSeq 6000 platform at BerryGenomics (Beijing, China) with a 400 bp insert size and a pair-end 150 bp sequencing strategy. The sequence reads were first filtered with MitoZ 3.6 [23] at default parameters. Then, the remaining clean paired reads were assembled using GetOrganelle-1.7.7.1 [24]. The annotation of genes was performed by MitoZ and manually double-checked with Geneious 8.0.5 [25]. The composition of the mitochondrial genome was calculated with MEGA7 [26].

### 2.3. Phylogenetic Tree Construction and Pairwise Distance Estimation

The phylogenetic tree was constructed based on 13 mitochondrial protein-coding genes (PCGs, Appendix A). All the PCG sequences were extracted by the script, extract_genes.py (https://github.com/tjcreedy/biotools, accessed on 8 July 2024). After aligning each gene sequence with MAFFT v7.526 [26], all alignments were concatenated with PhyloSuite v1.2.2 [27]. Model determination for MrBayes (Bayesian inference, BI) was generated with ModelFinder [28]. The phylogenetic tree was reconstructed using MrBayes version 3.2.7a [1]. Two Markov chains in the Bayes phylogenetic tree ran simultaneously, totaling 600,000 generations. Samples were collected every 5000 generations, and the first 25% was discarded as burn-in. The phylogenetic trees were visualized with iDOL (https://itol.embl.de/, accessed on 15 July 2024). Pairwise distances of each two species were computed using the bootstrap method of 1000 replications, calculating the p-distance with MEGA7 [26], other detailed parameters and the results can be seen in Appendix B.

### 2.4. Molecular Species Delimitation

This research focused on the molecular species delimitation for the genus *Gloydius* (Appendix C). To conduct a molecular species delimitation, two distance-based methods (ABGD [20] and ASAP [27]) and two tree-based methods (GMYC [28] and bPTP [22]) were performed. The ABGD (Automatic Barcode Gap Discovery) is a convenient method for alignment-based species delimitation, it enables rapid classification of species, performing under the JC69 Jukes-Cantor model with relative gap width (X = 0.015). ASAP (Assemble Species by Automatic Partitioning) can automatically delineate species, reducing intervention and enhancing the objectivity and accuracy of species delimitation, performed at default settings. GMYC (Generalized Mixed Yule Coalescent) takes into account evolutionary processes, such as speciation and gene flow, to provide more precise species delimitation, with an ultrametric tree generated from MrBayes using multiple sequences per species. bPTP (Bayesian Poisson Tree Processes) delineate species by identifying temporal shifts between interspecific and intraspecific branches, offering high accuracy and resolution, executing 100,000 Markov chain Monte Carlo generations with a thinning of 100 and with 20% discarded as burn-in.

## 3. Results

### 3.1. The Composition of the Mitochondrial Genome

In this study, all *Gloydius* species contained a typical 37 genes (22 tRNA and 2 rRNA genes, and 13 PCGs, Appendix A). The five newly sequenced mitogenomes resembled the order in previous sequenced samples [1,2,3,4,13,16]. The gene rearrangement phenomenon is not present in this genus. The nucleotide compositions of these mitogenomes are shown in Table 2. These *Gloydius* species exhibited the same AT nucleotide bias: 58%. Moreover, these mitogenomes had both a positive AT skew (0.10–0.11) and a CG skew (0.37 to–0.38).

### 3.2. Phylogenetic Relationships

**Figure 2:**
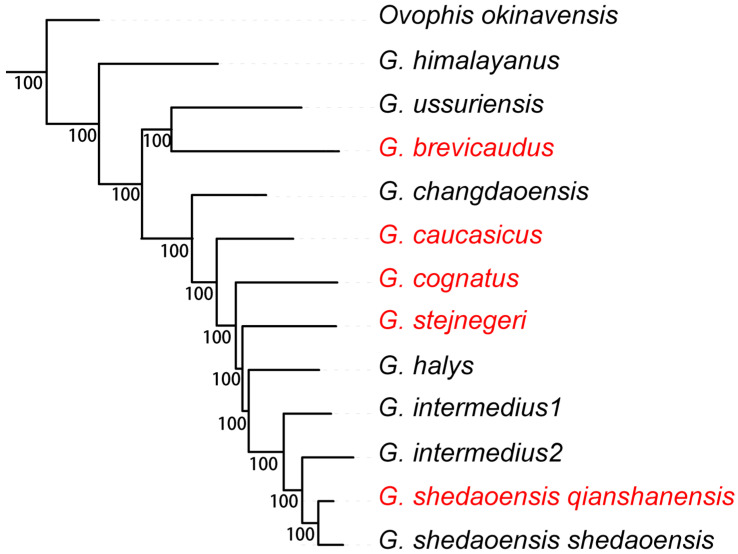
A MrBayes tree of *Gloydius* based on 13 mitochondrial PCGs. *O. okinavensis* was used as the outgroup. The numbers under branches indicate posterior probabilities, respectively. The red ones are newly added in this study.

The topology of the Bayesian inference (BI) tree displayed an identical cladogram with those in previous studies [1,2,3,4,7]. *Gloydius* species were clustered in a strongly supported monophyletic group with 100/100 posterior probabilities on all of the nodes (Figure 2). Figure 2 illustrates the Bayesian phylogenetic inference based on 13 mitochondrial protein-coding genes. The topology reveals a well-supported monophyletic lineage for the *Gloydius halys-intermedius* Complex, with strong Bayesian posterior probabilities at essential nodes. Notably, *G. caucasicus* and *G. stejnegeri* form distinct branches, supporting their divergence at specific level.

The phylogenetic position of *Gloydius himalayanus* from the southern slopes of the Himalayan ranges, is basal to, and considerably distant from, other species of *Gloydius* (p-distance: 11.7−13.2%). Nine samples that represent *G. halys-intermedius* are clustered in a monophyletic group sister to another monophyletic group comprising *G. brevicaudus* and *G. ussuriensis* (*G. blomhoffii* Complex). However, the samples of *G. intermedius* from South Korea did not form a monophyletic group with the samples from northeast China, as is mentioned by Lee et al. (2022) [19]. The four samples of four species, *G. caucasicus*, *G. cognatus*, *G. halys*, and *G. stejnegeri*, display paraphyly even though they were treated as subspecies of *G. halys* [5] or one single species in previous studies [10,12,18]. The taxonomic relationship between those clades will be discussed in the species delimitation section.

### 3.3. Species Delimitation

**Figure 3:**
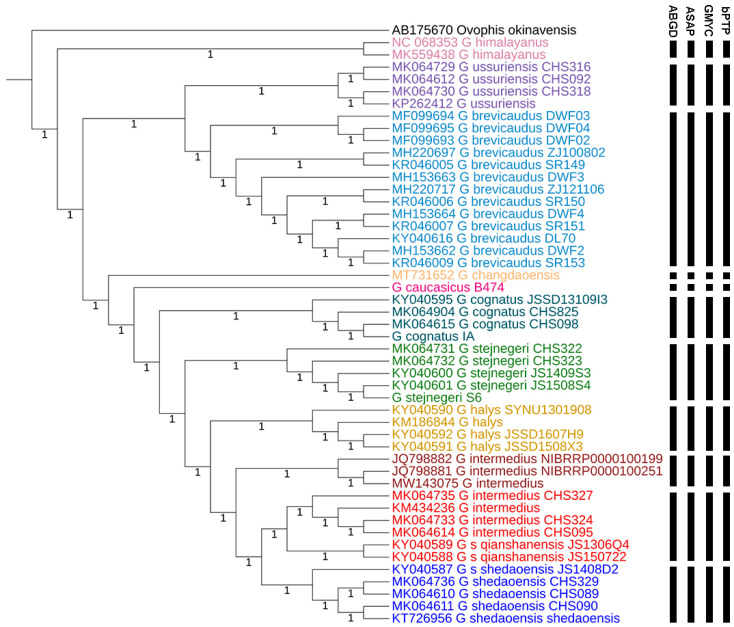
The molecular species delimitation of *Gloydius* based on COI gene with four methods (ABGD, ASAP, GMYC and bPTP). The samples delimitated as a single species are represented by a black bar. Every intermittent vertical black bar on the right side of the taxon name represents a single species determined by species delimitation (from left to right: ABCD, ASAP, GMYC, and bPTP). The numbers under branches indicate 100% posterior probabilities. Each color of the tips represents a species.

The results of specific delimitation by two distance-based methods (ABGD [20] and ASAP [27]) and two tree-based methods (GMYC [28] and bPTP [22]) are shown as vertical black bars (Figure 3). The summary of molecularly delimited species of all four approaches was identical: all four approaches revealed 11 molecular clades of *Gloydius* within the samples included in this study, and resemble the morphological species delimitation opinion [5,13].

The *G. halys-intermedius* Complex, *G. cognatus*, *G. caraganus*, *G. caucasicus*, *G. halys,* and *G. stejnegeri* each represent a valid specific taxon based on the species delimitation techniques. Note that the two populations of *G. intermedius* from northeast China and South Korea are determined as two distinct species (p-distance 2.1%), indicating cryptic diversity that requires further investigation.

## 4. Discussion

This study provides a higher-resolution molecular phylogenomic inference within the *G. intermedius-halys* Complex, based on the complete mtDNA genomes of the Sino-distributed species of genus *Gloydius* which face prolonged debate from different scientific publications. The specific-level taxonomic relationships within the *Gloydius halys-intermedius* Complex are clarified by both molecular phylogeny and specific delimitation models. The results correspond with previous morphological studies [5,13]. The complex situation of the taxonomy and phylogeny of the *G. halys-intermedius* Complex may be caused by the interspecific or intraspecies gene flows between different adjacent habitats. A further phylogenomic inference of *Gloydius* species with more samples included is required in order to investigate the origin, evolution, and migration of Asian pit vipers. The results indicate the specific-leveled genetic differentiation between the populations of *G. intermedius* from South Korea and northeast China. Further advanced species delimitation is encouraged to investigate this cryptic diversity.

Increasing the sample density and sequencing strength have provided a solid platform for species delimitation reviews, and phylogenomic inference construction. n globally widespread and complex species systems, the use of nuclear loci in phylogenomics is gaining increasing popularity. At the same time, sequencing known regions, combinations, and newly developed regions has become a trend in advanced species delimitation projects. The utility of mtDNA loci in molecular phylogenetics remains advantageous when it comes to accessibility, and many studies combine nucDNA and mtDNA when performing computations. Although nucDNA loci have been actively used in recent projects, mtDNA phylogenetic analysis remains essential for assessing systematics in complex biogeographical regions. While the trend encourages the combined use of mtDNA and nucDNA in constructing molecular phylogenomics, inferences based on mitochondrial genomes still provide a reference topology for subsequent topology comparisons.

## Figures and Tables

**Table 1:** Samples used in this study.

Vouchers	Species	Locations
AB175670	*Ovophis okinavensis* *	Japan, Okinawa Islands
B474	*G. caucasicus*	Azerbaijan, Lankaran Region
B6	*Gloydius brevicaudus*	Liaoning, China
IA	*G. cognatus*	Bayan Obo, Inner Mongolia, China
Q7	*Gloydius shedaoensis*	Wafangdian, Liaoning, China
S6	*Gloydius stejnegeri*	Lingshi, Shanxi, China
KM186844	*Gloydius halys*	Inner Mongolia, China
KM434236	*Gloydius intermedius* 2	Heilongjiang, China
KP262412	*Gloydius ussuriensis*	Heilongjiang, China
KT726956	*Gloydius shedaoensis*	Lvshun, Liaoning, China
MK559438	*Gloydius himalayanus*	Himachal Pradesh, India
MT731652	*Gloydius changdaoensis*	Changdao, Shandong, China
MW143075	*Gloydius intermedius* 1	Samcheok-si, Gangwon-do, South Korea

Note: * Outgroup.

**Table 2:** Nucleotide compositions of the whole mitogenomes of five newly sequenced *Gloydius* species. Both the AT skew and GC skew values from five species were positive, and the GC skew amplitude was greater than that of AT skew.

Samples	Species	Length /bp	T%	C%	A%	G%	AT Skew	CG Skew
B474	*G. caucasicus*	17,224	26.0	28.8	32.2	13.1	0.11	0.37
B6	*Gloydius brevicaudus*	16,655	26.1	28.5	32.2	13.2	0.10	0.37
IA	*G. cognatus*	17,228	25.9	28.8	32.4	12.9	0.11	0.38
Q7	*Gloydius shedaoensis*	17,216	25.8	28.9	32.4	12.9	0.11	0.38
S6	*Gloydius stejnegeri*	17,225	26.0	28.7	32.4	12.9	0.11	0.38

## Data Availability

All mitogenome sequences generated in this study were deposited in GenBank under accession numbers: PQ858434-PQ858438.

## Appendix A

**Table A1:** The abbreviations and full names of 13 mitochondrial Protein-Coding Genes.

Abbreviations	Full Names
*ND2*	NADH dehydrogenase subunit 2
*COX1*	cytochrome c oxidase subunit I
*COX2*	cytochrome c oxidase subunit II
*ATP8*	ATP synthase F0 subunit 8
*ATP6*	ATP synthase F0 subunit 6
*COX3*	cytochrome c oxidase subunit III
*ND3*	NADH dehydrogenase subunit 3
*ND4L*	NADH dehydrogenase subunit 4L
*ND4*	NADH dehydrogenase subunit 4
*ND5*	NADH dehydrogenase subunit 5
*ND6*	NADH dehydrogenase subunit 6
*CYTB*	cytochrome b
*ND1*	NADH dehydrogenase subunit 1

## Appendix B

### Appendix B.1

**Table A2:** The number of each species used for pairwise distances calculation.

No.	Species	Voucher Number
1	*G. caucasicus*	B474
2	*G. cognatus*	IA
3	*G. halys*	KM186844
4	*G. intermedius 2*	KM434236
5	*G. ussuriensis*	KP262412
6	*G. intermedius 1*	MW143075
7	*G. shedaoensis*	Q7
8	*G. stejnegeri*	S6
9	*G. brevicaudus*	B6
10	*G. himalayanus*	MK559438
11	*G. changdaoensis*	MT731652
12	*G. shedaoensis*	KT726956
13	*O. okinavensis*	AB175670-out

### Appendix B.2

**Table A3:** The pairwise distances of each two species of *Golydius*.

	1	2	3	4	5	6	7	8	9	10	11	12	13
1		0.005	0.005	0.004	0.009	0.005	0.005	0.004	0.008	0.008	0.005	0.005	0.009
2	0.045		0.005	0.005	0.009	0.005	0.005	0.005	0.008	0.008	0.005	0.005	0.01
3	0.037	0.037		0.004	0.009	0.004	0.004	0.004	0.009	0.009	0.005	0.005	0.009
4	0.036	0.04	0.026		0.009	0.003	0.003	0.004	0.009	0.008	0.005	0.003	0.009
5	0.108	0.113	0.112	0.112		0.009	0.009	0.009	0.008	0.008	0.009	0.009	0.009
6	0.039	0.042	0.026	0.021	0.114		0.004	0.004	0.009	0.008	0.005	0.004	0.009
7	0.035	0.044	0.03	0.015	0.114	0.026		0.005	0.009	0.008	0.005	0.003	0.009
8	0.033	0.041	0.031	0.033	0.114	0.034	0.038		0.008	0.009	0.005	0.005	0.009
9	0.116	0.123	0.125	0.127	0.106	0.119	0.129	0.119		0.009	0.009	0.009	0.009
10	0.114	0.118	0.125	0.121	0.114	0.122	0.117	0.119	0.132		0.008	0.008	0.008
11	0.042	0.054	0.051	0.047	0.109	0.049	0.046	0.048	0.118	0.115		0.006	0.009
12	0.042	0.049	0.036	0.019	0.112	0.029	0.011	0.044	0.127	0.123	0.052		0.009
13	0.137	0.137	0.139	0.14	0.128	0.137	0.137	0.139	0.147	0.12	0.129	0.14	

**Note:** Estimates of Evolutionary Divergence between Sequences. The number of base differences per site from between sequences are shown. Standard error estimate(s) are shown above the diagonal. Codon positions included were 1st + 2nd + 3rd. All positions containing gaps and missing data were eliminated. There were a total of 1602 positions in the final dataset. Evolutionary analyses were conducted in MEGA7 [26]. The values less than 0.03 were marked with pink background.

## Appendix C

**Table A4:** The samples used in this study for molecular species delimitation of *Golydius*.

Species	Voucher Number
*O. okinavensis* *	AB175670-Ovophis okinavensis
*G. shedaoensis*	MK064736_G._shedaoensis_CHS329
	MK064611_G._shedaoensis_CHS090
	MK064610_G._shedaoensis_CHS089
	KY040587_G._s_shedaoensis_JS1408D2
	KT726956_G._shedaoensis_shedaoensis
*G. intermedius*	MK064735_G_intermedius_CHS327
	MK064733_G_intermedius_CHS324
	MK064614_G_intermedius_CHS095
	KY040589_G_s_qianshanensis_JS1306Q4
	KY040588_G_s_qianshanensis_JS150722
	KM434236_G_intermedius
*G. stejnegeri*	MK064732_G_stejnegeri_CHS323
	MK064731_G_stejnegeri_CHS322
	KY040601_G_stejnegeri_JS1508S4
	KY040600_G_stejnegeri_JS1409S3
	G_stejnegeri_S6
*G. ussuriensis*	MK064730_G_ussuriensis_CHS318
	MK064729_G_ussuriensis_CHS316
	MK064612_G_ussuriensis_CHS092
	KP262412_G_ussuriensis
*G. brevicaudus*	MH220717_G_brevicaudus_ZJ121106
	MH220697_G_brevicaudus_ZJ100802
	MH153664_G_brevicaudus_DWF4
	MH153663_G_brevicaudus_DWF3
	MH153662_G_brevicaudus_DWF2
	MF099695_G_brevicaudus_DWF04
	MF099694_G_brevicaudus_DWF03
	MF099693_G_brevicaudus_DWF02
	KY040616_G_brevicaudus_DL70
	KR046009_G_brevicaudus_SR153
	KR046007_G_brevicaudus_SR151
	KR046006_G_brevicaudus_SR150
	KR046005_G_brevicaudus_SR149
*G. halys*	KY040592_G_halys_JSSD1607H9
	KY040591_G_halys_JSSD1508X3
	KY040590_G_halys_SYNU1301908
	KM186844_G_halys
*G. intermedius*	JQ798882_G_intermedius_NIBRRP0000100199
	JQ798881_G_intermedius_NIBRRP0000100251
	MW143075_G_intermedius
*G. changdaoensi*	MT731652_G_changdaoensis
*G. caucasicus*	G_caucasicus_B474

**Note:** The star * represents outgroup.
